# Evolution of inequalities in the coronavirus pandemics in Portugal: an ecological study

**DOI:** 10.1093/eurpub/ckab036

**Published:** 2021-03-16

**Authors:** Joana Alves, Patrícia Soares, João Victor Rocha, Rui Santana, Carla Nunes

**Affiliations:** 1 Escola Nacional de Saúde Pública, Universidade NOVA de Lisboa, Lisboa, Portugal; 2 NOVA National School of Public Health, Public Health Research Centre, Universidade NOVA de Lisboa, Lisboa, Portugal; 3 Comprehensive Health Research Center (CHRC), Lisboa, Portugal

**Keywords:** COVID-19, coronavirus, worst-affected areas, socioeconomic inequalities, disparities

## Abstract

**Background:**

Previous literature shows systematic differences in health according to socioeconomic status (SES). However, there is no clear evidence that the SARS-CoV-2 infection might be different across SES in Portugal. This work identifies the COVID-19 worst-affected municipalities at four different time points in Portugal measured by prevalence of cases, and seeks to determine if these worst-affected areas are associated with SES.

**Methods:**

The worst-affected areas were defined using the spatial scan statistic for the cumulative number of cases per municipality. The likelihood of being in a worst-affected area was then modelled using logistic regressions, as a function of area-based SES and health services supply. The analyses were repeated at four different time points of the COVID-19 pandemic: 1^st^ of April, 1^st^ of May, 1^st^ of June, and 1^st^ of July, corresponding to two moments before and during the confinement period and two moments thereafter.

**Results:**

Twenty municipalities were identified as worst-affected areas in all four time points, most in the coastal area in the Northern part of the country. The areas of lower unemployment were less likely to be a worst-affected area on the 1^st^ of April [AOR = 0.36 (0.14; 0.91)], on the 1^st^ of May [AOR = 0.03 (0.00; 0.41)], and on the 1^st^ of July [AOR = 0.40 (0.16; 1.05)].

**Conclusion:**

This study shows a relationship between being in a worst-affected area and unemployment. Governments and public health authorities should formulate measures and be prepared to protect the most vulnerable groups.

## 1. INTRODUCTION

In December 2019 an outbreak of a novel coronavirus began in the Chinese city of Wuhan. Later named SARS-CoV-2, the virus spread beyond China, and is currently the greatest pandemic experienced by the vast majority of this generation (1). On 11th of March 2020, the World Health Organization (WHO) declared COVID-19 a pandemic; two days later it was reported that Europe became the epicentre of the pandemic, with more cases reported on the European continent than in China. As of 20th of September more than 30 million cases were recorded and more than 950,000 deaths (2), causing substantial financial and societal impact worldwide (3).

There is little understanding of the distribution of health risk associated with COVID-19 across different socioeconomic levels. Previous literature shows systematic differences in health and mortality between persons with higher and lower socioeconomic position, whether measured by income, education, or occupation (4,5). Those differences are observed not only between the most and the least privileged but also in a gradient pattern, i.e., health deteriorates every step down the social position (6). The socioeconomic gradients are present in the health-related behaviours (such as smoking and drinking) and in the occurrence of health problems, disease, and ultimately death (6). Even more worrisome, this gradient is persistent, despite the health systems coverage and all the public health efforts. In most European countries inequalities have grown more accute over time, sometimes with large variations in magnitude (7).

In the context of the current coronavirus pandemic, there are some concerns that its incidence may not be equally distributed, and that it could exacerbate the existing health gap. This idea is not new, as historically pandemics have struck harder the poorest elements of society (8). In fact, from around the world new (as yet preliminary) data are emerging, revealing that some deprived communities and ethnicities are facing a greater disease burden and mortality (9–11).

Although the literature about the subject is scarce, we can hypothesize that some of the factors underlying the presence of socially patterned COVID-19 infection rates and deaths are common to the existence of socioeconomic inequalities in general health and mortality. During several stages of the disease, many inequalities can emerge: (i) the level of exposure may be different, so the risk of being infected would be socially patterned; (ii) the consequences of a positive diagnosis might be more severe in some groups than others; and (iii) the effects of the disease could be disproportionately distributed across society, and could remain long after the crisis has passed. These possibilities are further addressed below.

While the virus does not recognize a person's social stratum, persons' who are more socioeconomically deprived might be at greater risk of exposure. Individuals with lower education might have greater difficulties accessing information about SARS-CoV-2 infection, which could interfere with their understanding of preventive measures such as physical distancing and the proper use of a mask. They also may lack knowledge about where to go to obtain testing and recommendations for isolation. Education and literacy are important determinants of disease onset and have often been associated in research with the transmission of infectious diseases (12). Compliance with public health measures could be lower among less-educated individuals. Some authors argue that education is associated with differences in how individuals discount the future (13), suggesting that if one is more focused on gratification in the present, one might also be less willing to accept hardship linked to disease-prevention measures today. Also, according to economic theory, risk perception and aversion may differ according to education levels (14).

Adverse living conditions and poor neighbourhoods might be responsible for higher risk of infection. Higher population density, use of crowded public transportation, and overcrowded houses are important determinants of infectious diseases. Those determinants have previously been associated with infectious diseases, such as tuberculosis (15). Additionally, the strategies to reduce the risk of infection include good handwashing and hygiene, which are difficult in the absence of an adequate supply of running water and/or sanitation facilities at homes and workplaces. Overcrowding in the home might make self-isolation difficult or even impossible. Persons living in enclosed places in proximity to others, such as in nursing homes, prisons, or other correctional facilities, are of great concern. Under normal circumstances, these living conditions are already challenging in terms of public health, and they might enhance the transmission of the COVID-19 infection. Income inequality has also been previously linked to adverse health outcomes in a variety of ways, including the fact that poor persons will be at greater risk, and this could spill over to other socioeconomic groups, decreasing health for the society as a whole; and through the erosion of social cohesion, cooperation, and support (16).

Absence of job security, adequate pay, and social support might make it difficult to stay at home. People who work in non-specialized jobs might be more exposed to infection because they cannot maintain physical distance, they may be unable to work from home, or because they are subject to populated workplaces or must use overcrowded public transportation to commute to and from work. During the 2009 H1N1 pandemic, in the United States the incidence of influenza-like illness was associated with work-related inability to engage in physical distancing and household crowding, and these social factors were more prevalent among vulnerable populations such as Hispanics (17).

In sum, the literature suggests that SARS-CoV-2 exposure could have important social determinants. Also, the different measures implemented during the several stages of the pandemic to reduce personal contacts and slow the spread of the COVID-19 cases might have unequal effects across different socioeconomic groups. The idea that SARS-CoV-2 infection might be different across socioeconomic strata is especially worrying since those factors can exacerbate the socioeconomic differences in the long run, perpetuating health inequalities. In addition to being avoidable and unfair, in the long run health inequalities might contribute to rising healthcare and social security costs, and might even hinder economic growth (18,19). Understanding patterns of the disease would allow governments to devise measures and be prepared to protect the most vulnerable groups. Thus, this work identifies the COVID-19 worst-affected areas at four different time points in Portugal, measured by incidence of cases, and seeks to determine if these worst-affected areas are associated with socioeconomic factors. This will contribute to the research addressing the determinants of COVID-19 by studying the ecological-level social determinants of COVID-19 cases which, to the best of our knowledge have not previously been explored in Portugal.

## 2. Methods

We highlighted above that inequalities could emerge during exposure. However, inequalities might present different patterns at different time points. Our analyses will thus be structured according to those time points. Using different time points allows to capture if different measures implemented in the several stages of the pandemic had unintended effects across different socioeconomic groups.

Figure 1 shows the evolution of confirmed cases of COVID-19 in the first months of the pandemic. According to the Portuguese Directorate-General of Health (DGS), between the 1st of April and 1st of May, 17,000 new cases were recorded. During this same period, most European countries had implemented measures to reduce the spread of the disease. Portugal established a state of emergency and a nationwide lockdown on the 19th of March (*Decreto do Presidente da República* n. º 14-A/2020), and restrictions started to be gradually relaxed in May (*Decreto do Presidente da República*, n. º 20/2020; *Resolução do Conselho de Ministros* n. º 33-A/2020). By the end of that month Portugal had 32,000 confirmed cases. During June 10,000 additional cases of COVID-19 were confirmed in Portugal, representing a 30% increase in a single month, bringing the total number of cases to over 42,000. Our analysis therefore examines four points in time: April 1st (after the peak and during lockdown), May 1st (one month after lockdown), June 1st (one month after the end of lockdown), and July 1st (two months after the end of lockdown).

### 2.1. Data and variables

The number of cases was taken from the website of the Portuguese Directorate-General of Health (DGS) dedicated to the COVID-19. The explanatory variables were extracted from Statistics Portugal (INE, 2020). Since individual data on socioeconomic status is not available, we used municipality-based socioeconomic variables as a proxy of individual socioeconomic conditions and provide information about the municipality´s living conditions (20). The municipality-based socioeconomic conditions were proxied by income level, unemployment, and Gini coefficient since this was the information provided by Statistics Portugal at the municipality level. These variables reflect the baseline characterization of socioeconomic conditions, i.e., before the pandemic (pre-COVID-19). The variables used in the study and the sources are described in Table 1. The variables were measured at the municipal level.

### 2.2. Analysis

The authors first computed the cumulative number of COVID-19 cases per municipality at the four different time points under consideration. Then the worst-affected areas were identified using the spatial scan statistic at each time point. The dependent variable used by the spatial scan statistic was the number of COVID-19 cases divided by the population in each municipality. This methodology was proposed by Kulldorff (21) and tests the existence of significant spatial disease clusters. At each point the geographical units were Portuguese municipalities (n = 308). SaTScan™ software was used, and circular window shapes and isotonic spatial scan statistics were applied.

Then, crude and adjusted odds ratios were estimated for the likelihood of belonging to a worst-affected area using logistic regressions as a function of baseline area-based socioeconomic variables (pre-COVID-19) and health services supply. R version 3.6.2 was used for this purpose.

The analyses were repeated for all four time points of the pandemic: 1st of April, 1st of May, 1st of June, and 1st of July.

## 3. Results

Figure 2 indicates the location of the areas worst-affected by COVID-19 at the four time points of analysis. There were 74 municipalities identified at least once as a worst-affected area, and 20 of these were identified at all four time points. Most of the worst-affected areas were located in the northern coastal area of the country, including the municipality of Porto and the neighbouring municipalities (more details about the municipalities are in supplementary files 1 and 2). Lisbon, too, was considered a worst-affected area throughout the whole period of analysis, and in June and July some adjacent municipalities were also included in the worst-affected areas. Similarly, the municipality of Coimbra was included as a worst-affected area, with some adjacent municipalities included in June and July. By July there were 60 municipalities with COVID-19 rates significantly higher than the rest of the country. These included the 3 cases mentioned and a cluster of municipalities in the northernmost part of the country. The Alentejo region had single-municipality clusters during some periods, while the Algarve region revealed no worst-affected areas at the time points analysed.

Table 2 shows the crude odds ratio (OR) and adjusted odds ratio (AOR) for the logistic regressions using the worst-affected areas as the dependent variable (more details are in supplementary file 3). The likelihood of being in a worst-affected area was positively associated with the population density at the several time points [e.g. AOR=2.54 (1.61;4.02) on 1$^{st}$ of July]. The likelihood of being in a worst-affected area was generally associated with areas of higher unemployment rates on 1$^{st}$ of April [AOR= 0.36 (0.14;0.91)], on 1$^{st}$ of May [AOR=0.03 (0.00;0.41)], and on 1$^{st}$ of July [AOR=0.40 (0.16;1.05)]. The remaining variables were not significantly associated with the likelihood of being in a worst-affected area, and the adjusted odds ratio were not consistent across the several time points.

## 4. Discussion

Earlier research reports the existence of systematic differences in COVID-19 cases and mortality between people with higher and lower socioeconomic position in several countries (22,23). However, prior to our research there was no clear evidence that SARS-CoV-2 infection had different effects across the pre-existing socioeconomic groups in Portugal. We therefore undertook to identify the areas worst-affected by COVID-19, measured by incidence of cases, and test if those worst-affected areas revealed a socioeconomic pattern at several time points of the pandemic.

The results identify 20 municipalities as worst-affected areas at all four time points, most of which were in the northern coastal area of the country, including the municipality of Porto and neighbouring municipalities. This study shows an association between being in a worst-affected area and the unemployment rate and population density in that area, but not with the other socioeconomic variables, such as average earnings in the municipality or the inequality of the municipality. The areas of lower unemployment were less likely to be a worst-affected area on 1$^{st}$ of April [AOR= 0.36 (0.14;0.91)], on 1$^{st}$ of May [AOR=0.03 (0.00;0.41)], and on 1$^{st}$ of July [AOR=0.40 (0.16;1.05)]. The inclusion of some municipalities amongst worst-affected areas was greatly influenced by local outbreaks during this period, as reported by Moura and Reguengos de Monsaraz .

The likelihood of being in a worst-affected area was associated with higher unemployment rates. In the face of unemployment, people might seek other, informal income sources, and such work might expose them to greater risk of infection. The municipalities with higher unemployment are probably also the most deprived, with a large share of population lacking access to computers and/or internet connection, which are necessary for working from home. It is thus likely that these persons must leave home for work, and are more dependent on public transportation and more subjected to overcrowding conditions (24,25). This in turn hinders their ability to maintain social distancing and/or avoid populated places.

We were unable to obtain information regarding education levels across the municipalities. However, unemployment could reflect education levels in the municipality (26). People with higher education levels might seek more and higher quality of information about SARS-CoV-2 infection by selecting adequate sources of information. On the contrary, people with lower education could have difficulties understanding preventive measures, and how to access information about recommendations for isolation.

The association with population density was consistent across the four time points analysed. Without any restrictions, one may expect that more populated areas have a higher incidence of COVID-19 cases simply because areas with more people have a greater chance of crossing paths with an infected individual and spreading the disease. Similarly to other infectious diseases, population density might be an important factor in spreading the disease. People living in densely-populated areas might have difficulty with social distancing, even in times of lockdown.

We found no evidence of an association between being in a worst-affected area and earnings or inequalities by municipality. This is probably due to the fact that average earnings data and gini coefficient refer to full-time employees with full earning, omitting other types of income. Note that in Wuhan (China) areas with higher GDP per unit of land area were associated with lower rates of COVID-19 morbidity (27); in Barcelona districts with the lowest mean earnings had the highest incidence rates, while areas with highest mean earnings had the lowest incidence rates (28). Another explanation for the lack of association with monthly earnings and gini coefficient is the fact that those variables are aggregated by municipality. This could obscure the differences observed at the municipality-level. The variable “doctors per 1000 inhabitants” was only a proxy for access to testing, since we did not have those numbers per municipality. However, testing was not restricted to traditional healthcare facilities, which could explain the absence of significance.

In our findings, hospitalizations and mortality were associated with socioeconomic status. There was substantial variation in rates for COVID-19 hospitalizations across boroughs in New York City, with higher rates of hospitalization in regions with more people living in poverty and lower levels of educational attainment (29). Another study in the United States found evidence that in a nationwide perspective, states with higher income inequality had higher COVID-19 mortality rates (30). Unfortunately, we had no data for mortality per municipality to test for these relationships.

This study has some limitations. Individual-level data are not available. Thus, this study might suffer from ecological bias, i.e., attributing population characteristics to persons living in those geographical areas. Using municipal level as a unit of analysis might have hidden much of the variation within the municipalities. Additionally, the lack of information about the number of tests in each municipality is crucial. We assume that some municipalities have a higher incidence rate than others, simply reflecting the fact that more tests were performed, thereby skewing the incidence-rate data amongst municipalities. Due to data unavailability, we did not include in our analysis information that could have played important roles in the spred of the infection, such as proportion of people working in different economic sectors, mobility, and high tourist areas. Finally, the results of these analyses are largely influenced by the quality of the reported data. A recent study showed increasing measurement errors in three different datasets: two international (World Health Organization and European Centre for Disease Prevention and Control), and a national (Chinese Center for Disease Control and Prevention) (31). The measurement errors detected in these datasets included negative numbers and outliers on specific dates; variations in calendar date according to the occurrence, notification, and rrecording of cases leading to day lag between datasets; and differences by which cases were reported (either laboratory-confirmed, clinically diagnosed, or both) **(32).** It is understandable that errors occur, given the unprecedented context of the pandemic. Nonetheless, these jeapordize data comparability, as do variations depending on the dataset chosen.

These inequalities might not finish with the end of the pandemic. The consequences could be visible and pose problems for populations and researchers for many years. If the COVID-19 pandemic is inducing more inequality, as suspected, post-pandemic contexts could be even harsher for individuals with lower socioeconomic backgrounds. In a scenario of substantial economic slowdown, people who are already in the most fragile situations are going to be the most affected. For example, since people in precarious working conditions cannot work from home, with the implementation of social isolation measures and lockdown these individuals are unable to work during the pandemic. Individuals with lower education and poorer jobs are more likely to be subjected to lay-off measures and unemployment (25). Also, due to low income levels, they would likely have fewer savings to help them cope with expenditures during economic slowdown (32). Finally, being subject to physical distancing, isolation, uncertainty about the future, and lack of control of their lives, coupled with challenging economic situations (possibility of unemployment, reduced income levels) can increase stress and anxiety, which might introduce or exacerbate mental health issues amongst the most vulnerable groups. Several authors report a link between economic recessions and depression, alcoholism, and suicide rates (33,34). Previous economic crises have showed us that their impact on health depends on the protection given by social cohesion and by the social welfare state [17].

Understanding the patterns of disease allows governments to devise measures and be prepared to protect the most vulnerable groups. The analysis of geographic patterns of COVID-19, as performed in this study, is valuable to understand trends in transmission and assist decision-making processes; justifying why studies have been developed in different countries (35). Using the spatial scan statistic, a study in the United States identified emerging clusters, which should be prioritized in the implementation of measures and resource allocation (36). In Kuwait, intervention measures targeting vulnerable populations (migrant workers) lowered the number of clusters (37). In Equador, differences in COVID-19 mitigation strategies resulted in specific incidence and epidemic growth clusters (38). Clusters identified in South Korea were smaller than previous ones in both size and duration, providing evidence on the effectiveness of mitigation strategies (39). These studies illustrate the potential in identifying trends in COVID-19 transmission, worst-affected areas, and contextual factors. However, we need better information to monitor and evaluate those trends – namely more information regarding individual socioeconomic conditions, on a regular basis, and information on testing, at the neighbourhood level.

Earlier literature suggests that COVID-19 could have a socioeconomic pattern. This study confirms the existence of socioeconomic determinants in COVID-19 infection, finding an association between unemployment and COVID-19 incidence.

**Funding:** The present publication was funded by Fundação Ciência e Tecnologia, IP national support through CHRC (UIDP/04923/2020)..

**Conflict of interest:** there are no conflicts of interest to declare.

**Keypoints:**

- There were 20 municipalities identified as worst-affected areas for the cumulative number of cases at all four time points investigated.
- The areas of lower unemployment were less likely to be a worst-affected area.
- Health authorities should devise measures to be prepared to protect the most vulnerable groups.

**Table 1. Variables, sources and definitions.**

| Variable | Definition | Source |
|---|---|---|
| **COVID-19 cases** | | |
| Confirmed cases | Number of cumulative confirmed cases per day by municipalities | DGS, 2020 |
| **Demographics** | | |
| Population | Number of residents per municipality. | INE, 2018 |
| Older than 65 years old (%) | Percentage of residents more than 65 years old per municipality. | INE, 2018 |
| Male (%) | Percentage of male residents per municipality. | INE, 2018 |
| Population density (log) | Number of people resident per square kilometre in the Portuguese municipalities (Logarithm). | INE, 2018 |
| **Health services** | | |
| Doctors/1000 inhabitants | Number of registered doctors per municipality per 1000 inhabitants. | INE, 2018 |
| **Socioeconomic variables** | | |
| Unemployment | Number of persons registered as unemployed at the Portuguese public employment service (Instituto de Emprego e Formação Profissional, IEFP) divided by the number of residents (25 to 64 years old). | IEFP, 2017 |
| Earnings | Average monthly earnings by municipality (€). | INE, 2017 |
| Gini coefficient | Gini coefficient for the declared gross income, subtracted from the income tax. | INE, 2017 |

**Table 2. Odds ratio and adjusted odds ratio for the logistic regressions on the worst-affected areas.**

| | 1st April | | 1st May | | 1st June | | 1st July | |
|---|---|---|---|---|---|---|---|---|
| | OR (95%CI) | AOR (95%CI) | OR (95%CI) | AOR (95%CI) | OR (95%CI) | AOR (95%CI) | OR (95%CI) | AOR (95%CI) |
| **Demographics** | | | | | | | | |
| Older than 65 (%) | 0.93 (0.88;0.98) | 0.91 (0.81;1.02) | 0.82 (0.74;0.9) | 0.77 (0.59;1.00) | 0.88 (0.82;0.94) | 0.98 (0.86;1.13) | 0.93 (0.88;0.97) | 1.00 (0.89;1.12) |
| Male (%) | 0.75 (0.57;0.99) | 0.69 (0.40;1.20) | 0.86 (0.6;1.24) | 1.19 (0.29;4.94) | 0.90 (0.67;1.21) | 1.36 (0.74;2.48) | 0.71 (0.54;0.92) | 0.80 (0.46;1.38) |
| P.density (log) | 1.74 (1.41;2.15) | 1.38 (0.95;2.00) | 3.46 (2.34;5.13) | 3.24 (1.67;6.29) | 2.55 (1.92;3.38) | 2.95 (1.82;4.77) | 2.18 (1.73;2.74) | 2.54 (1.61;4.02) |
| **Health services** | | | | | | | | |
| **Nr. Doctors** | | | | | | | | |
| 1st tercile (-) | 0.23 (0.1;0.51) | 0.47 (0.14;1.50) | 0.08 (0.02;0.37) | 0.22 (0.02;2.65) | 0.18 (0.06;0.5) | 0.77 (0.16;3.65) | 0.22 (0.11;0.48) | 0.70 (0.22;2.23) |
| 2nd tercile | 0.41 (0.2;0.83) | 0.64 (0.24;1.74) | 0.17 (0.06;0.53) | 0.14 (0.02;1.13) | 0.47 (0.22;1.01) | 1.15 (0.36;3.67) | 0.35 (0.18;0.69) | 0.60 (0.22;1.64) |
| 3rd tercile (+) | 1.00 | 1.00 | 1.00 | 1.00 | 1.00 | 1.00 | 1.00 | 1.00 |
| **SES variables** | | | | | | | | |
| **Unemployment** | | | | | | | | |
| 1st tercile (-) | 0.27 (0.12;0.59) | 0.36 (0.14;0.91) | 0.06 (0.01;0.44) | 0.03 (0.00;0.41) | 0.71 (0.32;1.60) | 0.93 (0.31;2.80) | 0.28 (0.13;0.61) | 0.40 (0.16;1.05) |
| 2nd tercile | 0.36 (0.17;0.76) | 0.34 (0.15;0.81) | 0.50 (0.20;1.23) | 0.44 (0.12;1.61) | 0.59 (0.25;1.37) | 0.58 (0.20;1.73) | 0.55 (0.28;1.06) | 0.65 (0.28;1.52) |
| 3rd tercile (+) | 1.00 | 1.00 | 1.00 | 1.00 | 1.00 | 1.00 | 1.00 | 1.00 |
| **Earnings** | | | | | | | | |
| 1st tercile (-) | 0.88 (0.44;1.76) | 2.40 (0.78;7.41) | 0.35 (0.12;1.03) | 1.67 (0.29;9.76) | 0.32 (0.13;0.80) | 1.11 (0.33;3.73) | 0.84 (0.43;1.63) | 0.49 (0.18;1.30) |
| 2nd tercile | 0.52 (0.24;1.12) | 0.90 (0.34;2.41) | 0.43 (0.16;1.19) | 0.84 (0.20;3.61) | 0.59 (0.27;1.29) | 1.96 (0.69;5.59) | 0.57 (0.28;1.16) | 0.65 (0.29;1.47) |
| 3rd tercile (+) | 1.00 | 1.00 | 1.00 | 1.00 | 1.00 | 1.00 | 1.00 | 1.00 |
| **Gini coefficient** | | | | | | | | |
| 1st tercile (-) | 0.33 (0.15;0.73) | 0.50 (0.19;1.29) | 0.72 (0.26;2.02) | 3.24 (0.36;28.84) | 0.76 (0.30;1.91) | 1.17 (0.27;5.06) | 0.36 (0.17;0.77) | 4.71 (1.43;15.49) |
| 2nd tercile | 0.63 (0.31;1.25) | 0.56 (0.25;1.24) | 0.80 (0.30;2.18) | 2.78 (0.52;14.98) | 1.51 (0.67;3.41) | 1.49 (0.51;4.37) | 0.72 (0.38;1.38) | 1.71 (0.61;4.78) |
| 3rd tercile (+) | 1.00 | 1.00 | 1.00 | 1.00 | 1.00 | 1.00 | 1.00 | 1.00 |

**Legend:** (OR): unadjusted odds ratio; (AOR): adjusted odds ratio for the full model, including all socioeconomic variables and adjusted for demographics (more than 65 years old (%), male (%), population density (log), and the number of doctors (detailed results are in supplementary file 3)

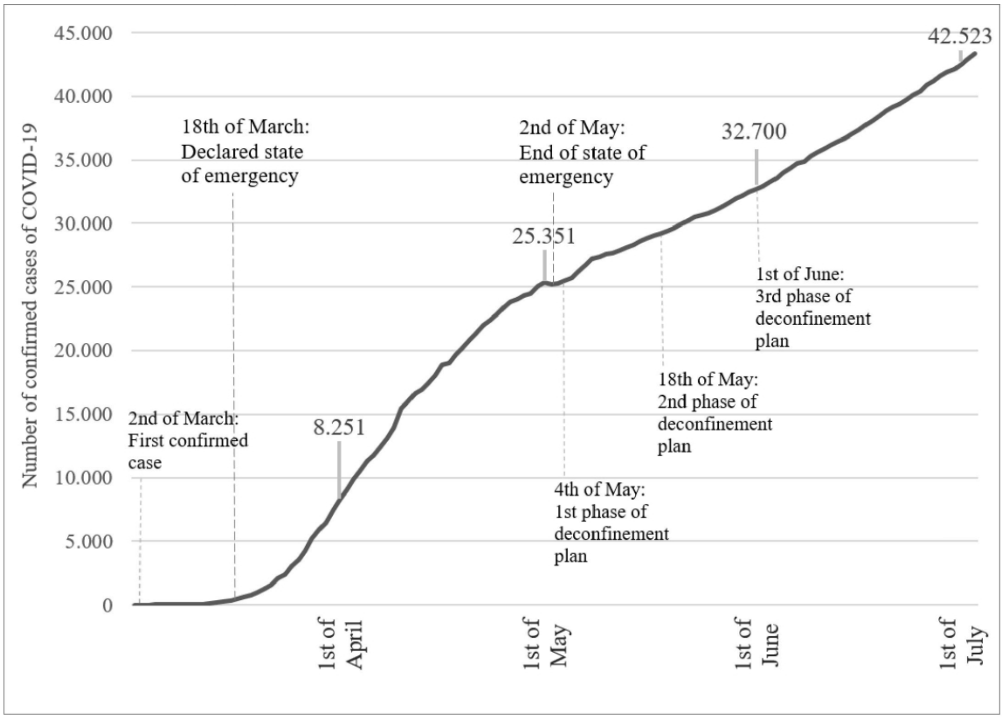

Evolution of the number of confirmed cases of COVID-19 in Portugal, 2020 (Source: Portugal Directorate-General of Health).

92x65mm (600 x 600 DPI)

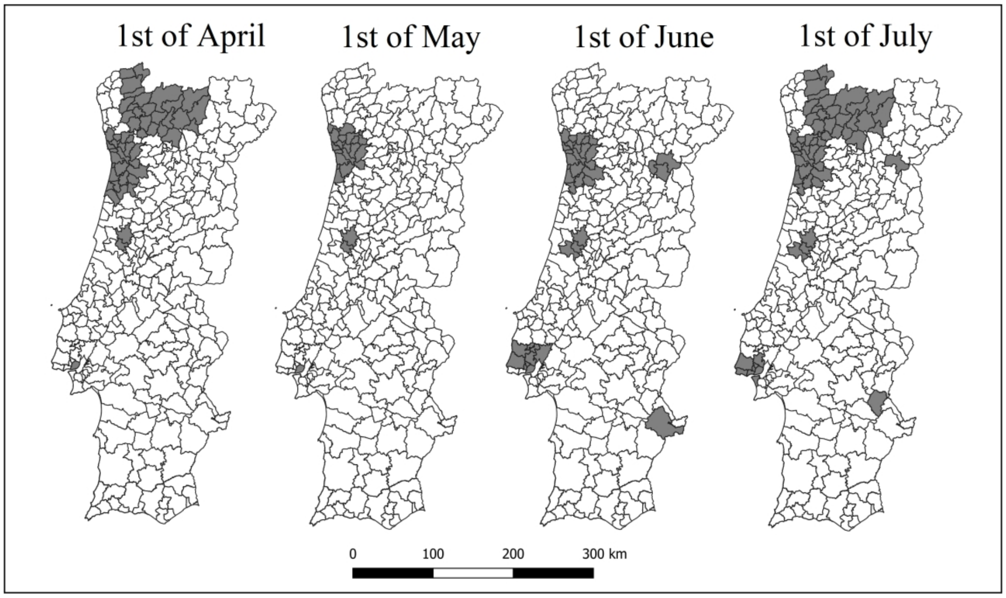

Geographic distribution of worst-affected areas of COVID-19, Portugal 2020.

310x183mm (600 x 600 DPI)
