# Supplementary material for: Evolution of inequalities in the coronavirus pandemics in Portugal: an ecological study
Source: Eur J Public Health. 2021 Mar 16;31(5):1069–75. doi: 10.1093/eurpub/ckab036 (PMC7989252; doi:10.1093/eurpub/ckab036)
Supplement: ckab036_Supplementary_Data [file ckab036_supplementary_data.zip › ejph-2020-09-om-1179-File004.docx]

**SUPPLEMENTARY FILE 1. Municipalities identified as worst-affected areas**

| **Time** | **Municipalities** |
| --- | --- |
| **1^st^ April** | Espinho, Vila Nova de Gaia, Santa Maria da Feira, Ovar, São João da Madeira, Gondomar, Porto, Oliveira de Azeméis, Matosinhos, Valongo, Estarreja, Murtosa, Castelo de Paiva, Maia, Paredes, Vale de Cambra, Arouca, Penafiel, Albergaria-a-Velha, Trofa, Sever do Vouga, Vila do Conde, Paços de Ferreira, Santo Tirso, Aveiro, Lisboa, Coimbra, Condeixa-a-Nova, Montalegre, Boticas, Vieira do Minho, Cabeceiras de Basto, Terras de Bouro, Ribeira de Pena, Chaves, Vila Pouca de Aguiar, Ponte da Barca, Póvoa de Lanhoso, Fafe, Melgaço, Mondim de Basto, Amares, Celorico de Basto, Arcos de Valdevez, Valpaços, Vila Verde, Guimarães, Murça, Vila Real, Braga, Monção, Felgueiras |
| **1^st^ May** | Porto, Matosinhos, Maia, Vila Nova de Gaia, Gondomar, Valongo, Espinho, Trofa, Paredes, Vila do Conde, Santo Tirso, Santa Maria da Feira, Paços de Ferreira, Penafiel, Vila Nova de Famalicão, Póvoa de Varzim, Castelo de Paiva, São João da Madeira, Ovar, Lousada, Vizela, Lisboa, Coimbra, Condeixa-a-Nova |
| **1^st^ June** | Vila Nova de Gaia, Gondomar, Espinho, Porto, Santa Maria da Feira, Valongo, Matosinhos, Maia, Paredes, São João da Madeira, Ovar, Castelo de Paiva, Penafiel, Trofa, Oliveira de Azeméis, Paços de Ferreira, Santo Tirso, Vila do Conde, Arouca, Vale de Cambra, Lousada, Estarreja, Loures, Odivelas, Amadora, Lisboa, Arruda dos Vinhos, Sobral de Monte Agraço, Vila Franca de Xira, Mafra, Sintra, Coimbra, Condeixa-a-Nova, Soure, Mêda, Penedono, Vila Nova de Foz Côa, Moura |
| **1^st^ July** | Loures, Odivelas, Amadora, Lisboa, Oeiras, Sintra, Almada, Cascais, Vila Nova de Gaia, Gondomar, Espinho, Porto, Santa Maria da Feira, Valongo, Matosinhos, Maia, Paredes, São João da Madeira, Ovar, Castelo de Paiva, Penafiel, Trofa, Oliveira de Azeméis, Paços de Ferreira, Santo Tirso, Vila do Conde, Arouca, Vale de Cambra, Lousada, Estarreja, Montalegre, Boticas, Vieira do Minho, Cabeceiras de Basto, Terras de Bouro, Ribeira de Pena, Chaves, Vila Pouca de Aguiar, Ponte da Barca, Póvoa de Lanhoso, Fafe, Melgaço, Mondim de Basto, Amares, Celorico de Basto, Arcos de Valdevez, Valpaços, Vila Verde, Guimarães, Murça, Vila Real, Braga, Monção, Felgueiras, Vizela, Reguengos de Monsaraz, Coimbra, Condeixa-a-Nova, Soure, Vila Nova de Foz Côa |
