# Supplementary material for: Evolution of inequalities in the coronavirus pandemics in Portugal: an ecological study
Source: Eur J Public Health. 2021 Mar 16;31(5):1069–75. doi: 10.1093/eurpub/ckab036 (PMC7989252; doi:10.1093/eurpub/ckab036)
Supplement: ckab036_Supplementary_Data [file ckab036_supplementary_data.zip › ejph-2020-09-om-1179-File006.docx]

**SUPPLEMENTARY FILE 3. Odds ratio and adjusted odds ratio for the logistic regressions on the worst-affected areas across several time points.**

**TABLE A. Odds ratio and adjusted odds ratio for the logistic regressions on the worst-affected areas, April 1^ST^.**

| **​** | **1st April** | | | | | | | | | | | |
| --- | --- | --- | --- | --- | --- | --- | --- | --- | --- | --- | --- | --- |
| **​** | **OR** | **(95% CI)** | **AOR** | **(95% CI)** | **AOR** | **(95% CI)** | **AOR** | **(95% CI)** | **AOR** | **(95% CI)** | **AOR** | **(95% CI)** |
| Demographics​ |  |  |  |  |  |  |  |  |  |  |  |  |
| Older than 65 yo (%)​ | 0.93 | (0.88;0.98) | 0.99 | (0.91;1.08) | 0.92 | (0.82;1.02) | 1.00 | (0.92;1.09) | 0.99 | (0.91;1.09) | 0.91 | (0.81;1.02) |
| Male (%)​ | 0.75 | (0.57;0.99) | 0.81 | (0.57;1.16) | 0.63 | (0.38;1.05) | 0.95 | (0.66;1.37) | 0.79 | (0.51;1.20) | 0.69 | (0.40;1.20) |
| Population density (log)​ | 1.74 | (1.41;2.15) | 1.58 | (1.16;2.13) | 1.34 | (0.96;1.86) | 1.84 | (1.32;2.56) | 1.54 | (1.11;2.15) | 1.38 | (0.95;2.00) |
| Health services |  |  |  |  |  |  |  |  |  |  |  |  |
| Number of doctors |  |  |  |  |  |  |  |  |  |  |  |  |
| 1^st^ tercile (lowest)​ | 0.23 | (0.1;0.51) | 0.57 | (0.22;1.45) | 0.55 | (0.20;1.51) | 0.33 | (0.12;0.94) | 0.66 | (0.25;1.77) | 0.47 | (0.14;1.50) |
| 2^nd^ tercile​ | 0.41 | (0.2;0.83) | 0.75 | (0.34;1.65) | 0.76 | (0.33;1.76) | 0.49 | (0.20;1.18) | 0.86 | (0.38;1.93) | 0.64 | (0.24;1.74) |
| 3^rd^ tercile (highest)​ | 1.00 |  | 1.00 |  | 1.00 |  | 1.00 |  | 1.00 |  | 1.00 |  |
| SES variables ​ |  |  |  |  |  |  |  |  |  |  |  |  |
| Unemployment​ |  |  |  |  |  |  |  |  |  |  |  |  |
| 1^st^ tercile (lowest)​ | 0.27 | (0.12;0.59) |  |  | 0.25 | (0.11;0.58) |  |  |  |  | 0.36 | (0.14;0.91) |
| 2^nd^ tercile​ | 0.36 | (0.17;0.76) |  |  | 0.29 | (0.13;0.66) |  |  |  |  | 0.34 | (0.15;0.81) |
| 3^rd^ tercile (highest)​ | 1.00 |  |  |  | 1.00 |  |  |  |  |  | 1.00 |  |
| Earnings​ |  |  |  |  |  |  |  |  |  |  |  |  |
| 1^st^ tercile (lowest)​ | 0.88 | (0.44;1.76) |  |  |  |  | 4.52 | (1.64;12.44) |  |  | 2.40 | (0.78;7.41) |
| 2^nd^ tercile​ | 0.52 | (0.24;1.12) |  |  |  |  | 1.42 | (0.57;3.56) |  |  | 0.90 | (0.34;2.41) |
| 3^rd^ tercile (highest)​ | 1.00 |  |  |  |  |  | 1.00 |  |  |  | 1.00 |  |
| Gini coefficient​ |  |  |  |  |  |  |  |  |  |  |  |  |
| 1^st^ tercile (lowest)​ | 0.33 | (0.15;0.73) |  |  |  |  |  |  | 0.49 | (0.20;1.20) | 0.50 | (0.19;1.29) |
| 2^nd^ tercile​ | 0.63 | (0.31;1.25) |  |  |  |  |  |  | 0.65 | (0.31;1.35) | 0.56 | (0.25;1.24) |
| 3^rd^ tercile (highest)​ | 1.00 |  |  |  |  |  |  |  | 1.00 |  | 1.00 |  |
| ROC |  |  | 0.75 | (0.67;0.82) | 0.79 | (0.72;0.86) | 0.78 | (0.70;0.85) | 0.75 | (0.69;0.82) | 0.81 | (0.75;0.87) |
| AIC |  |  | 258 | | 232.8 | | 251.8 | | 257.5 | | 232.2 | |

**TABLE B. Odds ratio and adjusted odds ratio for the logistic regressions on the worst-affected areas, May 1^ST^.**

| **​** | **1st May** | | | | | | | | | | | |
| --- | --- | --- | --- | --- | --- | --- | --- | --- | --- | --- | --- | --- |
| **​** | **OR** | **(95% CI)** | **AOR** | **(95% CI)** | **AOR** | **(95% CI)** | **AOR** | **(95% CI)** | **AOR** | **(95% CI)** | **AOR** | **(95% CI)** |
| Demographics​ |  |  |  |  |  |  |  |  |  |  |  |  |
| Older than 65 yo (%)​ | 0.82 | (0.74;0.9) | 0.88 | (0.75;1.02) | 0.77 | (0.60;0.98) | 0.89 | (0.76;1.05) | 0.87 | (0.74;1.03) | 0.77 | (0.59;1.00) |
| Male (%)​ | 0.86 | (0.6;1.24) | 1.32 | (0.68;2.56) | 1.27 | (0.34;4.71) | 1.54 | (0.70;3.39) | 1.30 | (0.57;2.98) | 1.19 | (0.29;4.94) |
| Population density (log)​ | 3.46 | (2.34;5.13) | 3.32 | (1.94;5.67) | 3.09 | (1.64;5.80) | 4.06 | (2.25;7.30) | 3.24 | (1.87;5.62) | 3.24 | (1.67;6.29) |
| Health services |  |  |  |  |  |  |  |  |  |  |  |  |
| Number of doctors |  |  |  |  |  |  |  |  |  |  |  |  |
| 1^st^ tercile (lowest)​ | 0.08 | (0.02;0.37) | 0.52 | (0.09;3.07) | 0.45 | (0.06;3.34) | 0.17 | (0.02;1.52) | 0.36 | (0.06;2.37) | 0.22 | (0.02;2.65) |
| 2^nd^ tercile​ | 0.17 | (0.06;0.53) | 0.35 | (0.09;1.46) | 0.28 | (0.05;1.50) | 0.12 | (0.02;0.74) | 0.28 | (0.06;1.25) | 0.14 | (0.02;1.13) |
| 3^rd^ tercile (highest)​ | 1.00 |  | 1.00 |  | 1.00 |  | 1.00 |  | 1.00 |  | 1.00 |  |
| SES variables ​ |  |  |  |  |  |  |  |  |  |  |  |  |
| Unemployment​ |  |  |  |  |  |  |  |  |  |  |  |  |
| 1^st^ tercile (lowest)​ | 0.06 | (0.01;0.44) |  |  | 0.04 | (0.00;0.37) |  |  |  |  | 0.03 | (0.00;0.41) |
| 2^nd^ tercile​ | 0.50 | (0.2;1.23) |  |  | 0.34 | (0.10;1.20) |  |  |  |  | 0.44 | (0.12;1.61) |
| 3^rd^ tercile (highest)​ | 1.00 |  |  |  | 1.00 |  |  |  |  |  | 1.00 |  |
| Earnings​ |  |  |  |  |  |  |  |  |  |  |  |  |
| 1^st^ tercile (lowest)​ | 0.35 | (0.12;1.03) |  |  |  |  | 11.62 | (1.67;80.76) |  |  | 1.67 | (0.29;9.76) |
| 2^nd^ tercile​ | 0.43 | (0.16;1.19) |  |  |  |  | 3.30 | (0.77;14.24) |  |  | 0.84 | (0.20;3.61) |
| 3^rd^ tercile (highest)​ | 1.00 |  |  |  |  |  | 1.00 |  |  |  | 1.00 |  |
| Gini coefficient​ |  |  |  |  |  |  |  |  |  |  |  |  |
| 1^st^ tercile (lowest)​ | 0.72 | (0.26;2.02) |  |  |  |  |  |  | 2.37 | (0.58;9.63) | 3.24 | (0.36;28.84) |
| 2^nd^ tercile​ | 0.80 | (0.3;2.18) |  |  |  |  |  |  | 1.00 | (0.29;3.48) | 2.78 | (0.52;14.98) |
| 3^rd^ tercile (highest)​ | 1.00 |  |  |  |  |  |  |  | 1.00 |  | 1.00 |  |
| ROC |  |  | 0.94 | (0.91;0.97) | 0.96 | (0.93;0.99) | 0.95 | (0.93;0.98) | 0.94 | (0.91;0.97) | 0.96 | (0.94;0.99) |
| AIC |  | | 110.16 | | 90 | | 106.8 | | 112.1 | | 95.4 | |

**TABLE C. Odds ratio and adjusted odds ratio for the logistic regressions on the worst-affected areas, June 1^ST^.**

| **​** | **1st June** | | | | | | | | | | | |
| --- | --- | --- | --- | --- | --- | --- | --- | --- | --- | --- | --- | --- |
| **​** | **OR** | **(95% CI)** | **AOR** | **(95% CI)** | **AOR** | **(95% CI)** | **AOR** | **(95% CI)** | **AOR** | **(95% CI)** | **AOR** | **(95% CI)** |
| Demographics​ |  |  |  |  |  |  |  |  |  |  |  |  |
| Older than 65 yo (%)​ | 0.88 | (0.82;0.94) | 1.04 | (0.93;1.16) | 0.97 | (0.86;1.11) | 1.04 | (0.93;1.17) | 1.05 | (0.94;1.19) | 0.98 | (0.86;1.13) |
| Male (%)​ | 0.90 | (0.67;1.21) | 1.26 | (0.84;1.90) | 1.28 | (0.72;2.27) | 1.34 | (0.87;2.06) | 1.47 | (0.86;2.52) | 1.36 | (0.74;2.48) |
| Population density (log)​ | 2.55 | (1.92;3.38) | 2.90 | (1.92;4.39) | 2.59 | (1.69;3.95) | 3.13 | (2.02;4.86) | 3.20 | (2.05;4.99) | 2.95 | (1.82;4.77) |
| Health services |  |  |  |  |  |  |  |  |  |  |  |  |
| Number of doctors |  |  |  |  |  |  |  |  |  |  |  |  |
| 1^st^ tercile (lowest)​ | 0.18 | (0.06;0.5) | 0.98 | (0.28;3.44) | 0.94 | (0.25;3.49) | 0.76 | (0.20;2.89) | 0.74 | (0.18;3.05) | 0.77 | (0.16;3.65) |
| 2^nd^ tercile​ | 0.47 | (0.22;1.01) | 1.36 | (0.51;3.63) | 1.27 | (0.47;3.44) | 1.04 | (0.36;3.03) | 1.28 | (0.46;3.56) | 1.15 | (0.36;3.67) |
| 3^rd^ tercile (highest)​ | 1.00 |  | 1.00 |  | 1.00 |  | 1.00 |  | 1.00 |  | 1.00 |  |
| SES variables ​ |  |  |  |  |  |  |  |  |  |  |  |  |
| Unemployment​ |  |  |  |  |  |  |  |  |  |  |  |  |
| 1^st^ tercile (lowest)​ | 0.71 | (0.32;1.6) |  |  | 0.80 | (0.30;2.11) |  |  |  |  | 0.93 | (0.31;2.80) |
| 2^nd^ tercile​ | 0.59 | (0.25;1.37) |  |  | 0.54 | (0.19;1.48) |  |  |  |  | 0.58 | (0.20;1.73) |
| 3^rd^ tercile (highest)​ | 1.00 |  |  |  | 1.00 |  |  |  |  |  | 1.00 |  |
| Earnings​ |  |  |  |  |  |  |  |  |  |  |  |  |
| 1^st^ tercile (lowest)​ | 0.32 | (0.13;0.8) |  |  |  |  | 1.99 | (0.56;7.00) |  |  | 1.11 | (0.33;3.73) |
| 2^nd^ tercile​ | 0.59 | (0.27;1.29) |  |  |  |  | 1.94 | (0.70;5.37) |  |  | 1.96 | (0.69;5.59) |
| 3^rd^ tercile (highest)​ | 1.00 |  |  |  |  |  | 1.00 |  |  |  | 1.00 |  |
| Gini coefficient​ |  |  |  |  |  |  |  |  |  |  |  |  |
| 1^st^ tercile (lowest)​ | 0.76 | (0.3;1.91) |  |  |  |  |  |  | 1.35 | (0.42;4.36) | 1.17 | (0.27;5.06) |
| 2^nd^ tercile​ | 1.51 | (0.67;3.41) |  |  |  |  |  |  | 2.08 | (0.76;5.68) | 1.49 | (0.51;4.37) |
| 3^rd^ tercile (highest)​ | 1.00 |  |  |  |  |  |  |  | 1.00 |  | 1.00 |  |
| ROC |  |  | 0.83 | (0.75;0.92) | 0.84 | (0.77;0.92) | 0.84 | (0.76;0.92) | 0.85 | (0.78;0.92) | 0.86 | (0.79;0.93) |
| AIC |  | | 182.7 | | 175.5 | | 184.2 | | 174.41 | | 172.7 | |

**TABLE D. Odds ratio and adjusted odds ratio for the logistic regressions on the worst-affected areas, July 1^ST^.**

| **​** | **1st July** | | | | | | | | | | | |
| --- | --- | --- | --- | --- | --- | --- | --- | --- | --- | --- | --- | --- |
| **​** | **OR** | **(95% CI)** | **AOR** | **(95% CI)** | **AOR** | **(95% CI)** | **AOR** | **(95% CI)** | **AOR** | **(95% CI)** | **AOR** | **(95% CI)** |
| Demographics​ |  |  |  |  |  |  |  |  |  |  |  |  |
| Older than 65 yo (%)​ | 0.93 | (0.88;0.97) | 1.06 | (0.96;1.16) | 0.98 | (0.88;1.1) | 1.08 | (0.98;1.18) | 1.07 | (0.97;1.17) | 1.00 | (0.89;1.12) |
| Male (%)​ | 0.71 | (0.54;0.92) | 0.84 | (0.58;1.22) | 0.66 | (0.40;1.10) | 1.03 | (0.70;1.52) | 0.85 | (0.56;1.30) | 0.80 | (0.46;1.38) |
| Population density (log)​ | 2.18 | (1.73;2.74) | 2.42 | (1.67;3.49) | 2.13 | (1.44;3.17) | 3.19 | (2.08;4.90) | 2.49 | (1.67;3.70) | 2.54 | (1.61;4.02) |
| Health services |  |  |  |  |  |  |  |  |  |  |  |  |
| Number of doctors |  |  |  |  |  |  |  |  |  |  |  |  |
| 1^st^ tercile (lowest)​ | 0.22 | (0.11;0.48) | 0.87 | (0.35;2.16) | 0.97 | (0.36;2.63) | 0.43 | (0.15;1.18) | 1.08 | (0.41;2.82) | 0.70 | (0.22;2.23) |
| 2^nd^ tercile​ | 0.35 | (0.18;0.69) | 0.85 | (0.39;1.88) | 0.91 | (0.39;2.11) | 0.45 | (0.18;1.12) | 0.99 | (0.44;2.25) | 0.60 | (0.22;1.64) |
| 3^rd^ tercile (highest)​ | 1.00 |  | 1.00 |  | 1.00 |  | 1.00 |  | 1.00 |  | 1.00 |  |
| SES variables ​ |  |  |  |  |  |  |  |  |  |  |  |  |
| Unemployment​ |  |  |  |  |  |  |  |  |  |  |  |  |
| 1^st^ tercile (lowest)​ | 0.28 | (0.13;0.61) |  |  | 0.23 | (0.10;0.56) |  |  |  |  | 0.40 | (0.16;1.05) |
| 2^nd^ tercile​ | 0.55 | (0.28;1.06) |  |  | 0.46 | (0.20;1.02) |  |  |  |  | 0.65 | (0.28;1.52) |
| 3^rd^ tercile (highest)​ | 1.00 |  |  |  | 1.00 |  |  |  |  |  | 1.00 |  |
| Earnings​ |  |  |  |  |  |  |  |  |  |  |  |  |
| 1^st^ tercile (lowest)​ | 0.84 | (0.43;1.63) |  |  |  |  | 8.14 | (2.71;24.46) |  |  | 0.49 | (0.18;1.30) |
| 2^nd^ tercile​ | 0.57 | (0.28;1.16) |  |  |  |  | 2.38 | (0.89;6.35) |  |  | 0.65 | (0.29;1.47) |
| 3^rd^ tercile (highest)​ | 1.00 |  |  |  |  |  | 1.00 |  |  |  | 1.00 |  |
| Gini coefficient​ |  |  |  |  |  |  |  |  |  |  |  |  |
| 1^st^ tercile (lowest)​ | 0.36 | (0.17;0.77) |  |  |  |  |  |  | 0.47 | (0.19;1.14) | 4.71 | (1.43;15.49) |
| 2^nd^ tercile​ | 0.72 | (0.38;1.38) |  |  |  |  |  |  | 0.72 | (0.34;1.53) | 1.71 | (0.61;4.78) |
| 3^rd^ tercile (highest)​ | 1.00 |  |  |  |  |  |  |  | 1.00 |  | 1.00 |  |
| ROC |  |  | 0.78 | (0.71;0.85) | 0.82 | (0.76;0.88) | 0.81 | (0.75;0.88) | 0.78 | (0.72;0.85) | 0.84 | (0.78;0.90) |
| AIC |  | | 253.4 | | 231.4 | | 240.9 | | 253.6 | | 229 | |
