# Supplementary figures and images for: Evolution of inequalities in the coronavirus pandemics in Portugal: an ecological study
Source: Eur J Public Health. 2021 Mar 16;31(5):1069–75. doi: 10.1093/eurpub/ckab036 (PMC7989252; doi:10.1093/eurpub/ckab036)

Supplementary file 2. Geographic distribution of socioeconomic variables for Portugal.

| 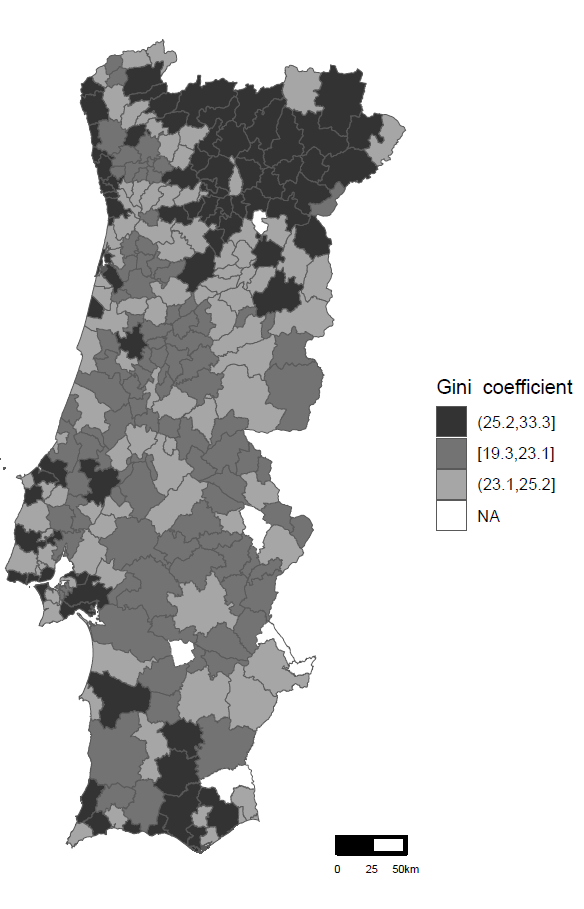  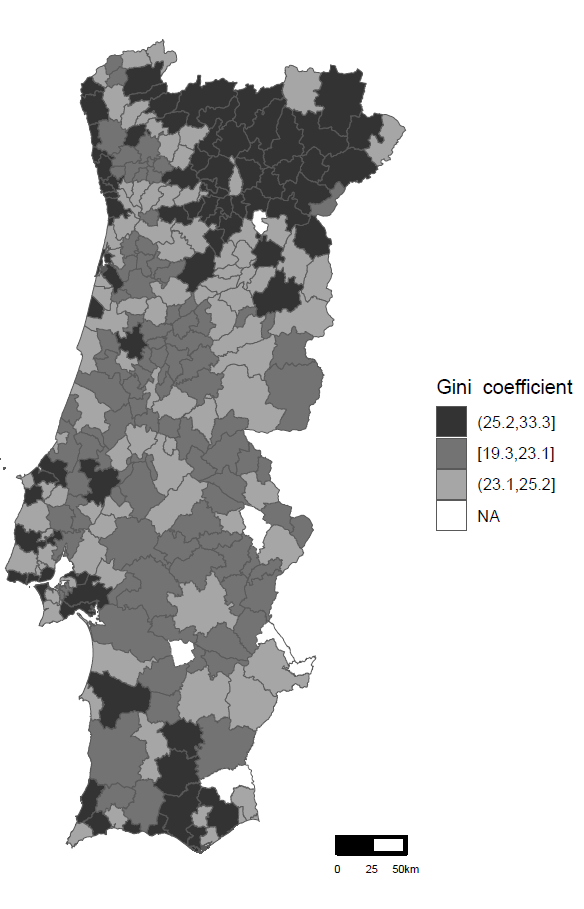 | 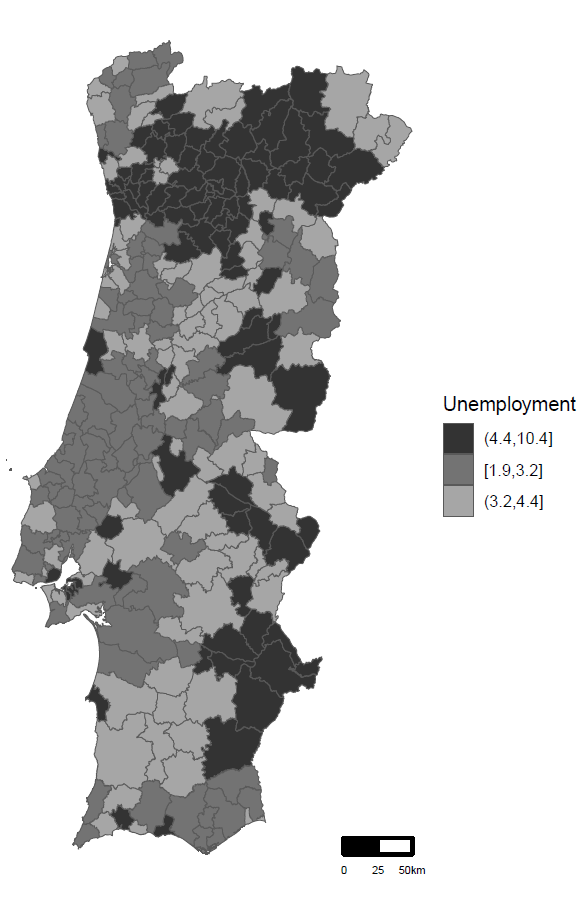  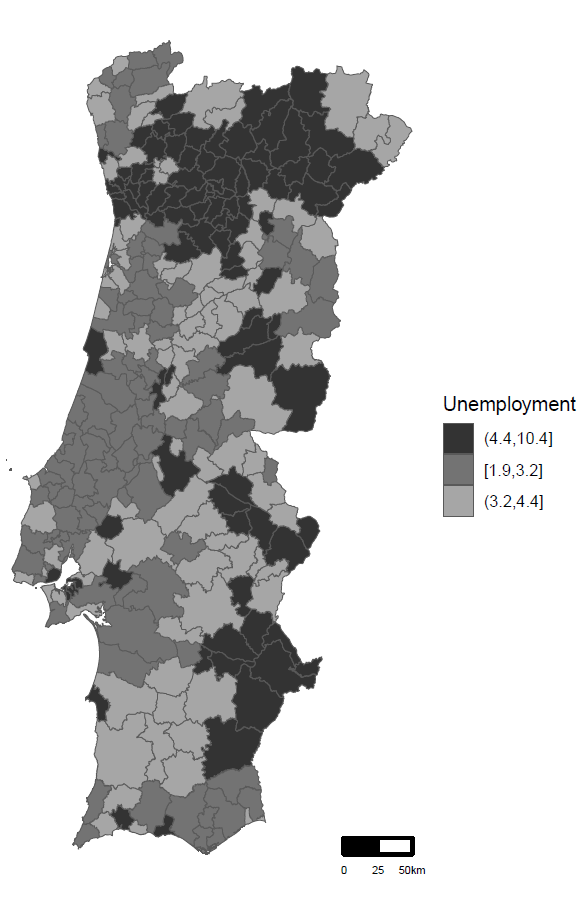 | 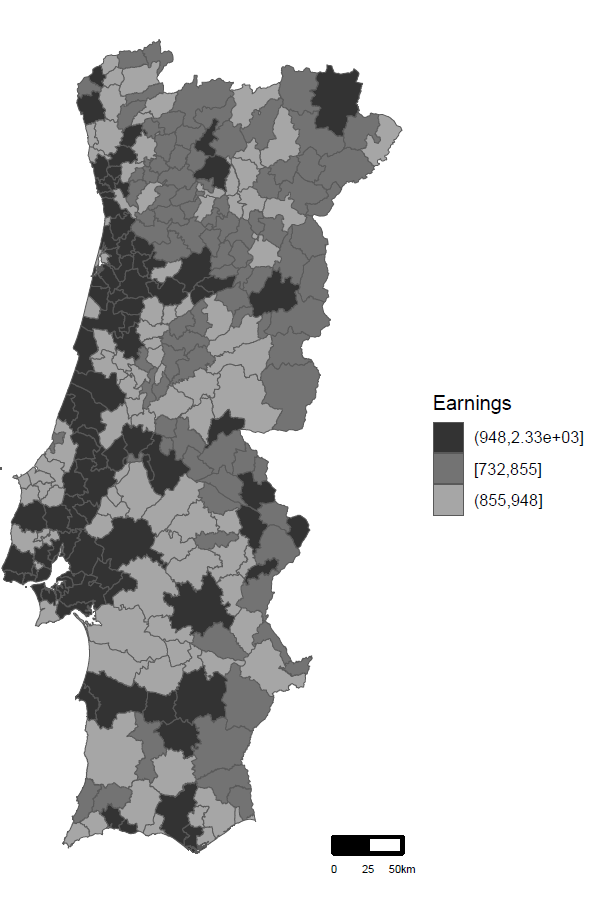  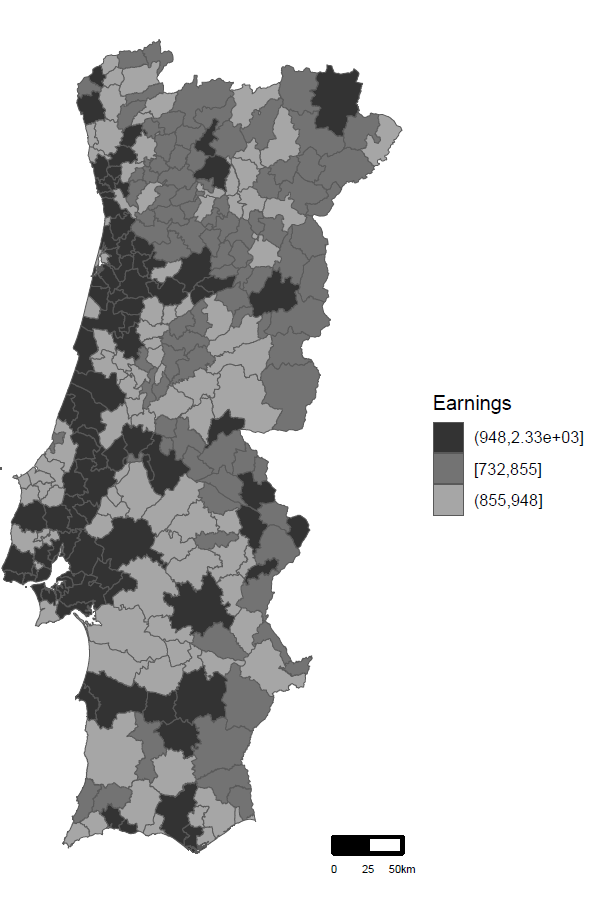 | 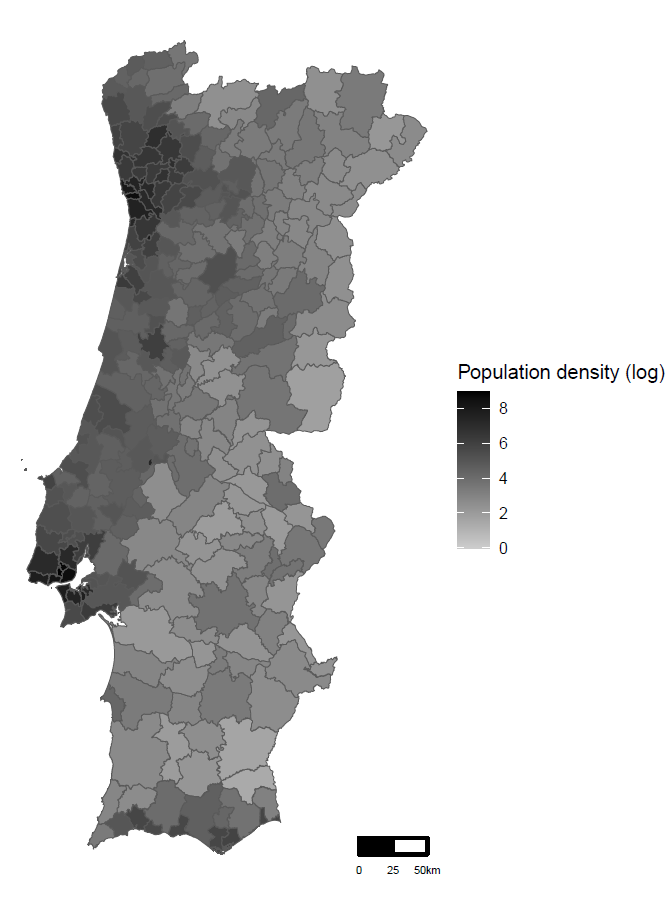  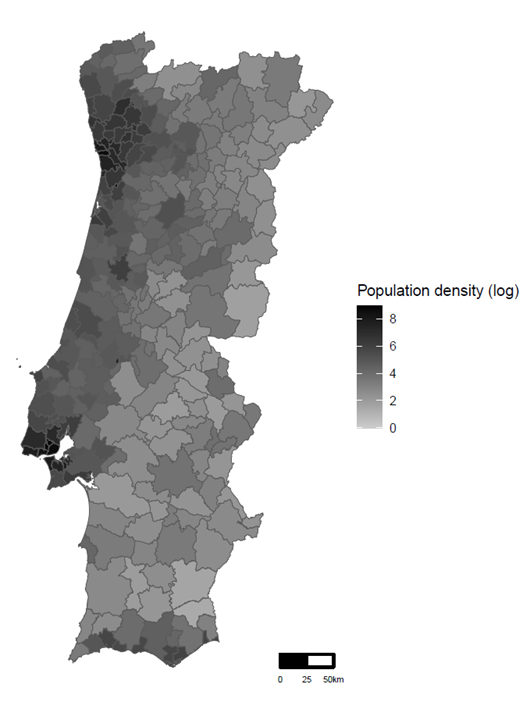 |
| --- | --- | --- | --- |

Supplement: ckab036_Supplementary_Data [file ckab036_supplementary_data.zip › ejph-2020-09-om-1179-File005.docx]
